# Eph Receptors and Ephrins in Retinal Diseases

**DOI:** 10.3390/ijms22126207

**Published:** 2021-06-08

**Authors:** Radoslaw Kaczmarek, Pawel Gajdzis, Malgorzata Gajdzis

**Affiliations:** 1 Department of Ophthalmology, Wroclaw Medical University, 50-556 Wroclaw, Poland; radoslaw.kaczmarek@umed.wroc.pl; 2 Department of Pathomorphology and Oncological Cytology, Wroclaw Medical University, 50-556 Wroclaw, Poland; pawel.gajdzis@umed.wroc.pl

**Keywords:** Eph, Eph receptors, ephrin, retina, diabetic retinopathy, proliferative vitreoretinopathy, retinopathy of prematurity, age-related macular degeneration, choroidal neovascularization

## Abstract

Retinal diseases are the leading cause of irreversible blindness. They affect people of all ages, from newborns in retinopathy of prematurity, through age-independent diabetic retinopathy and complications of retinal detachment, to age-related macular degeneration (AMD), which occurs mainly in the elderly. Generally speaking, the causes of all problems are disturbances in blood supply, hypoxia, the formation of abnormal blood vessels, and fibrosis. Although the detailed mechanisms underlying them are varied, the common point is the involvement of Eph receptors and ephrins in their pathogenesis. In our study, we briefly discussed the pathophysiology of the most common retinal diseases (diabetic retinopathy, retinopathy of prematurity, proliferative vitreoretinopathy, and choroidal neovascularization) and collected available research results on the role of Eph and ephrins. We also discussed the safety aspect of the use of drugs acting on Eph and ephrin for ophthalmic indications.

## 1. Introduction

Membrane-bound ephrin receptors (Eph) are the largest subpopulation of tyrosine kinase receptors (RTKs) [1]. They are found in similar forms in all vertebrates and some invertebrates [2]. It has been shown that the more complex an organism is, the more Eph receptors it has, which may indicate their role in the organization and structure of the organism [3,4]. This hypothesis seems to be confirmed by the high complexity of the interactions involving the Ephs.

**Figure 1:**
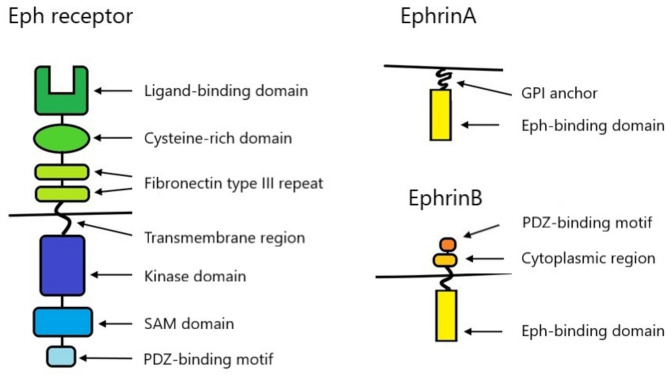
Structure of Eph and ephrins.

Due to the structure and the ability to bind ligands, Eph are divided into two subtypes—EphA (consisting of 9 receptors—EphA1–A8, EphA10) and EphB (consisting of 5 receptors—EphB1–B4, EphB6). All Eph types contain a single transmembrane domain. The glycosylated extracellular fragment contains a ligand-binding domain (also responsible for receptor dimerization and clustering), a cysteine-rich domain, and two repeats of type III fibronectin motifs. The intracellular fragment consists of a tyrosine kinase domain, a SAM (sterile α motif) domain, and a PDZ-binding motif. The presence of the SAM domain is a unique feature distinguishing Eph in the RTK receptor group. It participates in interactions between receptors, supporting homo- or heterotypic oligomerization, and regulates the process of receptor-dependent endocytosis [1]. Figure 1 shows the structure of Eph schematically. The ligands for Eph are ephrins—proteins associated with cell membranes, also divided into two subclasses—ephrinA (A1–A6) and ephrinB (B1–B3) [1]. EphA binds preferentially to ephrinsA and EphB to ephrinsB. There are exceptions like EphA4, which binds to both ephrinsA and ephrinsB, or ephrinA5, which binds not only to EphA but also to EphB2 [2,5]. EphrinsA are tethered to the extracellular cell membrane via a glycosylphosphatidylinositol (GPI) anchor. EphrinsB are transmembrane proteins with a short cytoplasmic region containing a PDZ-binding motif (Figure 1) [1].

Most tyrosine kinase receptors are activated by dimerization. Activating Eph requires the formation of more organized groups—at least tetramerization—to achieve the full effect [2,5]. However, reactions with smaller or larger groups are observed, and the effect can be enhanced by higher aggregation [5,6]. Eph can hetero-oligomerize within the subtypes—for example, EphB1 and EphB4 can form functional complexes with EphB6, and also between subtypes—for example, EphA3 can react with EphB2 [7,8]. Recent studies also show that Eph can respond with other RTK receptors and other transmembrane receptors [9,10,11]. To activate reaction, Eph must be in a trans configuration. It then combines with ephrin on an adjacent cell. If Eph is in the cis configuration, it can bind to ephrin on the same cell and this reaction can stop the trans reaction effect [12,13]. Moreover, only membrane-bound or artificially grouped ephrins can activate the Eph transmission process, while ungrouped soluble ephrins act as Eph antagonists. Soluble forms of Eph can also act as antagonists.

The above-described relationships show a high degree of complexity of the reactions in which Eph and ephrins participate. However, the most unique feature of the Eph is the ability to activate bidirectional signaling [5]. It results from the ability to activate signaling pathways in both receptor-expressing cells and ligand-containing cells, and the effects of these reactions may be opposite [14]. Signaling leads to the modification of the actin cytoskeleton and the organization of microtubules by control of intracellular proteins and the expression of surface adhesion molecules, thereby regulating cell attraction and repulsion, migration, and invasion capacity [15].

Due to its unique properties, Eph has become the subject of intensive research in recent years, especially in the context of cancer. Their role in the pathogenesis of autoimmune and degenerative diseases, atherosclerosis, Alzheimer’s, Parkinson’s, and amyotrophic lateral sclerosis is also being investigated. In this study, we focused on the known of Eph and ephrins in retinal diseases associated with the abnormal blood vessels’ formation and fibrosis. It is a diverse group of diseases, with different pathogeneses, that is often related to vision-threatening complications, causing severe problems in treatment.

## 2. Angiogenesis

The essence of angiogenesis is the formation of new branches from already existing blood vessels. The process is initiated by ischemia or hypoxia. Angiogenic factors activate endothelial cells and weaken the connections between them and smooth muscle cells in the vessel walls. The endothelial cells then proliferate and migrate towards the ischemic area, and form tubular structures. The final stage is the maturation of new vessels, involving the recruitment of pericytes and stabilization of the extracellular matrix [16]. Angiogenesis processes are essential for the proper functioning of the body. They enable wound healing, reperfusion after ischemic injuries and changes during the menstrual cycle and pregnancy. They can often also be associated with pathological processes such as tumor vascularization, changes in the course of diabetes or atherosclerosis [17,18].

One of the most important and well-studied regulators of angiogenesis are tyrosine kinase (RTK) receptors, in particular vascular endothelial growth factor (VEGF) receptors. These receptors, when activated by hypoxia, trigger multiple signaling pathways leading to proliferation, migration, survival, and vascular permeability during angiogenesis [19]. Other RTK receptors involved in the angiogenesis process are vascular-specific Tie-2 and Tie-1, platelet-derived growth factor (PDGF) receptors and Eph receptors. The Eph/ephrin signaling system is actively involved in the migration of endothelial cells, in a mechanism very similar to the guidance of neurons [20].

Eph and ephrins are bound to cell membranes. To initiate the angiogenesis process, an additional factor is needed that activates Eph and ephrins in response to ischemia in tissues initially distant from the existing blood vessels. One hypothesis is that vascular endothelial growth factor (VEGF) is that factor. Unbound to cell membranes, soluble VEGF, in response to hypoxia can initiate angiogenesis by activating endothelial cells and inducing their proliferation. Then, as vessels begin to grow into the ischemia area, the Eph–ephrin pathway is activated, causing vessel growth to stop and transform into fully functional structures [21]. VEGF is a particularly good starting point for therapy—especially in oncology and ophthalmology. The effectiveness of the administration of preparations that inhibit VEGF activity has been confirmed in many clinical trials, but there are still cases of no or poor response to treatment [22]. Therefore, the operation of VEGF-dependent signaling pathways remains the subject of numerous studies.

Chen et al., in studies on liver cancer cell lines, showed that switching off EphA1 causes a significant reduction in the expression of VEGF and MMP-2 and MMP-9 (matrix metalloproteinase). Since vascular density was significantly lowered, it was theorized that the decrease in VEGF and MMP expression was induced by angiogenesis inhibition [23]. Dobrzanski et al. found that the simultaneous blocking of Eph and VEGF signaling pathways was more effective in inhibiting the spread of endothelial cells (compared to blocking each of them separately) and, consequently, the formation of new vessels [24]. In their research, Cheng et al. presented considerable evidence for the relationship of the EphA with the process of VEGF-dependent angiogenesis. They proved that soluble Eph receptor inhibits VEGF-dependent angiogenesis but does not affect FGF-induced angiogenesis (fibroblast growth factor). They also showed that EphA activation is essential for VEGF-dependent endothelial cell migration. They also found that activation of EphA is necessary for endothelial (VEGF-dependent) survival but has no effect on their proliferation [25]. Groppa et al. found that EphB4 and ephrinB2 acted as regulators of VEGF-dependent proliferation of endothelial cells [26]. Du et al. also presented evidence linking the VEGF, EphB2, and ephrinB2 signaling pathways [27]. Generally, EphB4 and efrinB2 signaling systems are specific to the vascular system. They are thought to regulate the differentiation of arteries and veins, being less effective for the proliferation of endothelial cells [28]. The results published so far indicate that while VEGF is the most important factor inducing angiogenesis, the role of Eph and ephrins is also very important. They appear to have a primarily regulatory function by activating, inhibiting, and modeling VEGF-related signaling.

## 3. Stabilization of Vessel Walls

For the proper functioning of small blood vessels, stabilization of their walls is critical—a process dependent mainly on pericytes (PCs) and smooth muscle cells located in the vessel walls (vSMCs), as well as on their mutual relations. PCs mainly cover capillaries, while vSMCs are found in the walls of arteries, larger veins, and large lymph vessels. The normal, contact-dependent function of these cells is essential for the proper functioning of blood vessels and is in part controlled by the Eph/ephrin signaling pathways. In diabetic retinopathy, the loss of PCs leads to the formation of microaneurysms, a chaotic proliferation of immature vessels, macular edema, and blindness. Similarly, a small number or loose connections between them are associated with the presence of unorganized and leaky blood vessels within tumors, predisposing to edema and hemorrhage. vSMCs are involved in the pathogenesis of many vascular diseases, including arteriosclerosis, aneurysms, varicose veins, and hypertension, and in the event of injuries or surgeries, they may move and strengthen the wall of adjacent blood vessels [29,30]. Malfunctions in PCs and vSMCs function cause leakage of vessel walls, edema in surrounding tissues and hemorrhages.

Eph is known to affect the function of PC cells and vSMCs. EphB4/ephrinB2 signaling plays a key role in communication between PC and endothelial cells, particularly important in stabilizing vascular walls [31]. In experimental studies in mice, reducing Eph expression by switching off genes causes extensive skin hemorrhage, generalized fetal swelling, and death in the perinatal period. In the absence or significantly reduced expression of selected ephrins, in particular ephrin B2, PCs more loosely adhere to the vessel walls, not forming the uniform layer necessary for proper functioning. vSMCs also form more chaotic structures and additionally infiltrate lymph vessels in an unorganized way. Notably, the number of PCs and vSMCs remains normal, and only the function is impaired [29]. Deroanne et al. showed that EphA significantly influenced the proliferation of vSMCs and, through them regulate vascular wall tension and blood pressure [32]. The results obtained in that study suggest a relationship between Eph expression and the functioning of blood vessel walls, particularly their integrity and tightness.

## 4. Eph and Ephrin Expression Profile in Healthy Retina

The expression of Eph and ephrins within the retina is often analyzed in the context of the process of guiding neurons and developing the visual pathway in the embryonic period. Numerous studies have shown that the phenomenon of an expression gradient of selected Ephs and ephrins occurs in the retina and visual pathways, constituting a specific map for neurons [33]. So far, the role of numerous Eph receptors and ephrins in the retinal mapping process has been confirmed, including EphA3, EphA4, EphA5, EphA6, EphB1, EphB2, EphB3, EphB4, ephrinA2, ephrinA5, ephrinB1, and ephrinB2 [34,35,36]. For example, EphA6 is mainly responsible for the development of the macular area, reaching a peak of expression at the developing fovea. Simultaneously, during the development of the retina in the macular area, decreased expression of ephrinA1, -A2, -A3, and -A4 is found [37]. EphA6 is found in the ganglion cell layer, and expression varies both across the thickness of this layer and depending on the foveal and parafoveal versus peripheral locations [38]. Unfortunately, there are no cross-sectional studies of Eph and ephrins’ expression within the mature retina. Researchers focus only on single receptors and ligands, especially in various disease states. For this reason, little is known about the role of Eph and ephrin in a mature, healthy retina. Numerous studies are conducted in animal models and on tissues removed from human eyes during retinal repair surgery [18]. For example, the human retina has been shown to express EphB4 and ephrinB2, with ephrinB2 mediating endothelial cell migration and proliferation in the retina [39,40].

Research results also confirm that selected Ephs and ephrins are involved in retinal vascularization development. Kozulin et al. showed that EphA6, with their ligands ephrinA1 and -A4, play an essential role in the retinal vascular patterning [41]. Selected Ephs also influence the development of the optic nerve. They are responsible, among other things, for the proper closure of the optic fissure [42]. Moreover, they also play an important role in the glaucoma-related processes of apoptosis of the fibers that form the optic nerve [43,44]. Eph and ephrins are involved in the pathogenesis of many disorders within the ocular tissues. In this study, we will focus on selected retinal diseases related to neovascularization and fibroblast proliferation.

## 5. Diabetic Retinopathy

Diabetes is being diagnosed in an increasing number of patients. It is a chronic disease and patients are exposed to a number of serious complications, mainly related to the malfunctioning of small blood vessels. These complications not only reduce the quality of life but can also lead to severe disability and death (for example, kidney failure, myocardial infarction, or stroke).

**Figure 2:**
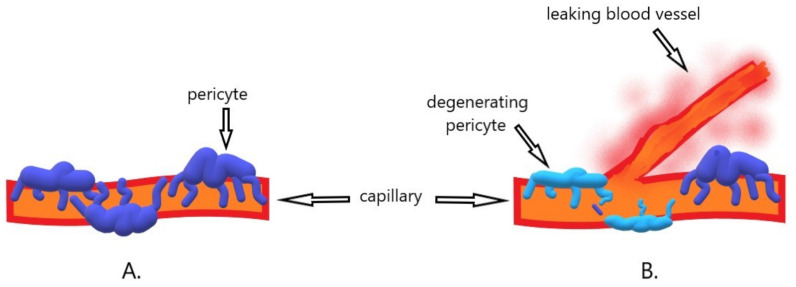
Schematic illustration of the mechanism of diabetic retinopathy. (**A**). The presence of pericytes ensures proper functioning and stability of capillary walls. (**B**). Due to the hypoxia of the retina and the expression of angiogenic factors, new branches of the already existing vessels are formed through angiogenesis. Due to the atrophy and degeneration of the pericytes, the capillary walls become fragile, which leads to leakage and then edema of the retina.

Diabetic retinopathy is one of the most serious ocular complications of diabetes. All diabetic patients are at risk of developing retinal complications, with the most important risk factors being disease duration, hyperglycemia, hypertension, and hyperlipidemia [45]. The excess persistent blood glucose is converted by the aldose reductase pathway, which converts sugars into alcohol. The sorbitol formed in this reaction damages the function of the pericytes [46]. This weakens the capillaries, leading to the formation of microaneurysms [47]. As a result of hyperperfusion initiated by high glucose concentration and exacerbated by high blood pressure, further damage to the blood vessel walls occurs. Eventually, the capillaries’ walls are closed, and the retina becomes hypoxic. Then the retina produces vasoproliferative factors that stimulate the reconstruction of the existing and the formation of new blood vessels [45]. Unfortunately, mainly because they lack pericyte support, the newly formed blood vessels are defective, leaky and break easily (Figure 2). They cause swelling and hemorrhages [31]. The role of VEGF in the process of angiogenesis is best understood and documented [48,49]. However, there are reports on the role of Eph and ephrins in the pathogenesis of diabetic retinopathy.

Participation in the EphA receptor signaling pathway is necessary to achieve the maximum effect of VEGF-induced neovascularization [25]. Following this lead, Mao et al. set out to see if ephrinA1 levels were associated with the symptoms of diabetic retinopathy. They observed that plasma levels of ephrinA1 were increased in patients with diabetic retinopathy. They also suggested that plasma ephrinA1 levels are a more sensitive biomarker detecting diabetic retinopathy than VEGF [50]. This discovery raises hope for new diagnostic possibilities in the early stages of diabetic retinopathy. This may be particularly useful in young patients and with co-existing kidney injury, where one of the basic diagnostic tests—fluorescein angiography—cannot be performed.

Ojima et al. investigated the effect of ephrinA1 administered by intravitreal injection on retinal neovascularization and VEGF-induced retinal vascular leakage in a diabetic retinopathy model [51]. They observed a significant reduction in neovascularization. As VEGF is the primary modulator of neovascularization in the rat model used in the study, they concluded that ephrinA1 is an inhibitor of VEGF. Intravitreal administration of ephrinA1 also reduced retinal vascular leakage in a dose-depending manner. It is especially important in the context of treating the early stages of retinopathy, where traditional laser therapy may be considered too radical (due to its possible side effects such as visual field limitation, scotopic vision deterioration, and the risk of bleeding).

The role of ephrinB2 in angiogenesis is well-understood. Du et al. found that the expression of ephrinB2 is increased in diabetic rats. Moreover, exposure of animals to hypoxia for 12 h significantly increases the expression of ephrinB2 [52]. Li et al., in turn, conducted studies on neovascular membranes obtained from patients with diabetes. It turned out that in comparison with healthy retinas (obtained from deceased donors) they show a higher expression of ephrinB2 and EphB4 [53].

Umeda et al. showed that ephrinB2, EphB2, and EphB3 were expressed in fibroproliferative membranes obtained from patients with diabetic retinopathy during vitrectomy (65%, 90% and 35% respectively). The vascular density in diabetic retinopathy tissues was significantly greater than that seen in retinopathy of prematurity tissues, where there was also much less expression of ephrinB2, EphB2, and EphB3 (25%, 70%, and 45% respectively). There was, however, no relationship between the expression of Ephs and clinical features [54]. The demonstrated difference in Eph expression indicates a slightly different mechanism underlying pathological changes in diabetic retinopathy and preterm retinopathy. Demonstration of Eph expression in proliferative membranes suggests that the receptors and their associated ephrins are involved in the pathogenesis of the disease. Perhaps by modulating the action of Eph and ephrins in an appropriate manner, it would be possible to treat severe retinal complications of diabetes.

A summary of the identified associations of Eph and ephrins with diabetic retinopathy is presented in Table 1.

## 6. Retinopathy of Prematurity (ROP)

**Figure 3:**
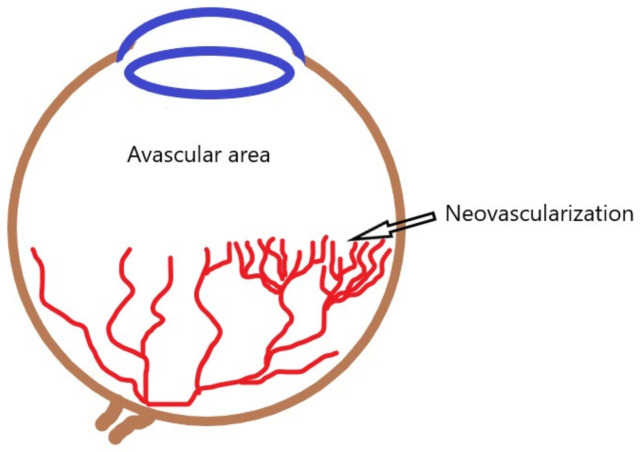
Schematic representation of preterm retinopathy. Blood vessel growth is inhibited by exposure to hyperoxia. The hypoxic peripheral avascular area of the retina produces angiogenic factors, leading to uncontrolled neovascularization.

Preterm infants have incompletely vascularized peripheral retina. After the state of physiological intrauterine hypoxia, they are exposed to hyperoxia (atmospheric oxygen pressure and oxygen supplementation) [55]. As a result of malnutrition, lack of growth factors, sepsis, and exposure to non-physiological oxygen concentrations, the existing ones become constricted, and the formation of new blood vessels in the retina is disturbed. In the second phase, the hypoxic retina produces angiogenic factors, which leads to uncontrolled neovascularization [56] (Figure 3).

The basic treatment methods are laser ablation of the ischemic retina and intravitreal injections of anti-VEGF preparations. In more advanced cases, a vitrectomy may be necessary [57]. The pathophysiology of ROP is relatively well-understood, and the early diagnosis and well-treated disease give a real chance of maintaining good vision in the future. However, a corresponding ROP experimental model for oxygen-induced retinopathy is relatively simple to create, and research into it can provide a lot of helpful information. And while it is unlikely that such studies will soon affect ROP management patterns, they may help treat other retinal diseases, such as diabetic retinopathy or proliferative vitreoretinopathy.

Vihanto et al. in their studies showed that hypoxia increased the expression of EphB4, ephrinB2, EphA2, and ephrinA1 within the skin [58]. This confirms the potential role of Eph and ephrins in hypoxic diseases. Further evidence comes from studies in animal models of ROP. Du et al. conducted a series of studies investigating the role of ephrinA4 in the murine ROP model [59]. They found that ephrinA4 is significantly overexpressed in postnatal days P13, P15 and P17 compared to healthy animals. In normal development, the expression of ephrinA4 was found only after P17. Moreover, ephrinA4 levels decreased on days P19 and P21 when there was spontaneous regression of neovascularization. The localization of expression also differed depending on the state of the retina, and standard ephrinA4 staining was identified in the retinal ganglion neuron layers and the inner nuclear layer, while in mice with retinopathy, an expression also appeared in the nerve fiber layer. To fully emphasize the role of ephrinA4 in neovascularization, studies have also shown that blocking of ephrinA4 with an intravitreal injection of an inhibitor markedly decreased the number of neovascular tufts, suppressing pathologic retinal neovascularization and reducing avascular areas. Very similar results were obtained in the studies on ephrinA5 [60]. In this case, it was also possible to demonstrate a membrane expression profile, particularly in endothelial cells.

Chen et al. used a newborn rat model of ROP to evaluate the effect of a soluble form of the EphA2 receptor, known to block EphA receptors, on the formation of blood vessels within the retina [61]. It turned out that blocking EphA receptors did not affect the formation of normal retinal vessels, but significantly reduced pathological neovascularization. Moreover, soluble EphA2 receptor inhibited ephrinA1 and VEGF-induced vascular endothelial cell migration. The presented results are extremely important in the context of the potential treatment of ROP. Laser therapy is an invasive procedure that causes irreversible destruction of retinal fragments. On the other hand, more recently used intravitreal anti-VEGF injections may delay the formation of normal blood vessels. They also often require repeated applications, increasing the risk of complications such as intraocular hemorrhage or endophthalmitis, and expose children to the need for further general anesthesia. The discovery of a drug that could inhibit pathological neovascularization while not disrupting and possibly even accelerating the formation of normal blood vessels would be a huge step forward in ROP therapy.

Zamora et al. investigated the effect of intravitreal administered soluble forms of EphB4 and ephrinB2 in the oxygen-induced retinopathy model [62]. It turned out that there was a 69% and 66% reduction in the pathologic pre-retinal tuft formation in the eyes after the injections, respectively. He et al. came to similar results in their research where soluble EphB4 inhibited choroidal NV in a model of laser-induced NV [63]. Also, Ehlken et al. showed that sEphB4 inhibited hypoxia-induced angiogenesis. Their research also proved the opposite effect, namely stimulation of EphB4 and ephrinB2 signaling enhanced hypoxia-induced angiogenesis. Based on the analysis of distinction in the expression kinetics, they concluded that EphB4 and ephrinB2 form two independent signal pathways, regulated in the opposite way. These results correspond to the idea of bidirectional signaling, so characteristic for Eph and ephrin [64]. However, stimulation of endogenous ephrinB2 with soluble EphB4 typically induces the migration and proliferation of endothelial cells [65,66]. Therefore, further research is needed to understand the mechanism of action of substances affecting Eph / ephrin signaling pathways for future therapeutic use. Unlike neovascular tufts, the receptors’ soluble forms had no effect on intraretinal vessel formation.

A summary of the identified associations of Eph and ephrins with ROP is presented in Table 2.

## 7. Proliferative Vitreoretinopathy (PVR)

**Figure 4:**
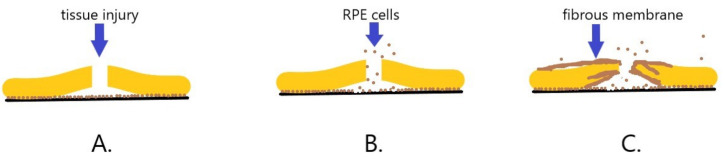
Diagram of the development of proliferative vitreoretinopathy (PVR). (**A**). As a result of tissue injury and retinal disruption, the neurosensory layer of the retina separates from the retinal pigment epithelium (RPE). (**B**). RPE cells migrate through the retinal break towards the vitreous chamber. (**C**). RPE cells transdifferentiate to fibroblasts and then begin to proliferate and produce collagen, forming fibrous membranes that cover and overgrow the retina.

Proliferative vitreoretinopathy (PVR) is a severe complication of retinal detachment. Despite intensive progress in vitreoretinal surgery, PVR remains the leading cause of treatment failure in cases of retinal detachment and penetrating injury of the eye. Regardless of the treatment, it can lead to blindness. PVR is a complex process, similar to wound healing, involving damage to ischemic tissue, inflammation, and the proliferation of several types of cells [67]. Fibroblasts seem to play the most significant role in PVR development. The separation of neuroretina from the underlying RPE causes tissue injury and triggers a cascade of events [68]. Cells from the RPE migrate to the vitreous chamber, transdifferentiate into fibroblasts or macrophages, and start to proliferate. This leads to the contraction of the cellular membrane, extracellular collagen production and creation of fixed folds in the retina. The structures most characteristic of PVR are formed-fibrous membranes. The membranes can extend over the retina’s inner and outer surfaces, generating traction, opening treated retinal breaks and creating new ones [67,68] (Figure 4). The only therapeutic option is to perform surgery, during which the membranes are mechanically removed, releasing the traction, and attaching the retina. Sometimes it is necessary to perform the operations repeatedly, but the disease continues to progress, worsening the patient’s vision. Research into the role of Eph in the PVR formation can help with treatment.

He et al. demonstrated the expression of EphB4 and ephrinB2 in early-passage human RPE cells and PVR membranes harvested during surgery, but not within the normal RPE monolayer cells [63]. Moreover, soluble EphB4 blocked EphB4 and ephrinB2 phosphorylation in RPE cells in vitro and limited migration and proliferation of RPE cells induced by PDGF. The effect depended on the dose of sEphB4 [69]. RPE cells after sEphB4 application show a reduction in FAK and p42/44 MAPK dependent phosphorylation. These are signaling pathways involved in PDGF-mediated proliferation and migration. These findings create a real chance to use EphB4/ephrinB2 signaling inhibitors for the treatment of PVR. EphrinB2 is generally involved in fibrosis in many organs, including the lungs and kidneys. In vivo studies showed that silencing EphrinB2 with lentiviral vector improves cardiac function and reduces fibrosis in mice, and blockade of ephrinB2 with imatinib reduces pathological sinusoidal remodeling in a mouse model of liver injury. Moreover, mice genetically lacking fibroblast expression of Ephrin-B2 show remarkable resistance to bleomycin-induced pulmonary fibrosis. The reduction of fibrosis demonstrated in the above studies by influencing Eph/ephrin signaling raises hope for the possibility of using this type of therapy in eyes with developing PVR [70].

A summary of the identified associations of Eph and ephrins with PVR is presented in Table 3.

## 8. Choroidal Neovascularization (CNV)

Choroidal neovascularization (CNV) is the pathological process underlying age-related macular degeneration (AMD), and also occurs in other eye diseases such as myopia, infections, inflammations, or injuries [71,72]. Theoretically, it can develop when the continuity of the Bruch’s membrane lying underneath the pigment epithelium is broken [73]. AMD is the leading cause of irreversible severe visual impairment worldwide. The studies conducted so far indicate that VEGF plays a crucial role in the pathogenesis of the neovascular form of AMD. It is also the main target of therapy—anti-VEGF preparations administered in the form of intravitreal injections. However, a certain group of patients does not respond to the therapy, or even develop resistance to the treatment, resulting in a reduced therapeutic effect [22]. In CNV diseases other than AMD, no specific therapeutic protocols have been developed. Anti-VEGF therapy is also used in these cases, not always with satisfactory outcomes.

**Figure 5:**
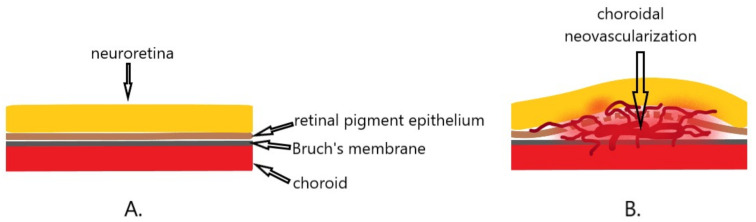
Diagram of choroidal neovascularization. (**A**). Normally, the Bruch’s membrane and the retinal pigment epithelium separate the neuroretina from the choroid. (**B**). Disturbances in homeostasis result in the formation of abnormal blood vessels that overgrow Bruch’s membrane and spread under the pigment epithelium and in the subretinal space, causing neuroretina elevation and swelling.

The essence of CNV is the disturbance of chorioretinal homeostasis, leading to the proliferation of blood vessels that surmount the Bruch’s membrane, then spreading under and through the retinal pigment epithelium (RPE) and under the subretinal space (Figure 5). In this process, RPE cells undergo transdifferentiation, proliferation and deposition in the CNV stroma [74].

Provis has suggested in his work that the macular area of the retina is much more prone to degenerative changes associated with neovascularization than any other region of the retina [75]. The reason is the anatomical structure. The macular center is adapted to the highest resolution vision. Cone photoreceptors reach their highest numerical density within the central fovea. Due to their intensive metabolism, they need much oxygen, and its source is the choroid, as there are no retinal vessels within the fovea. The absence of retinal blood vessels in the fovea allows for a better quality of central vision, but also lacks a functional reserve for oxygen supply. As a result, even the most minor disturbances related to vascular diseases, cardiovascular diseases, smoking, or diabetes, can cause metabolic stress and then the development of degenerative changes and neovascularization.

Martin et al., examining the expression of various angioregulatory factors in neovascular membranes obtained from AMD patients during vitrectomy, showed, that these membranes express EphA7 [76]. On the other hand, they did not show EphA7 expression in healthy RPE cells, which indicates their participation in the pathogenesis of CNV.

He et al. showed that the choroidal endothelial cell (CEC) express EphB4 and ephrinB2 [62]. Intravitreal injection of the soluble form of EphB4 inhibited CEC migration and reduced laser-induced CNV formation in animal models (rats). CNV membranes showed a reduction in leakage score in fluorescein angiography. Histologically, CNV membranes after injection of sEphB4 were smaller and showed reduced vascularity. In a further, more recent study, He et al. showed again that intravitreal injection of the soluble form of EphB4 reduces leakage from CNV membranes in fluorescein angiography and reduces CNV volume [77]. Moreover, the tissues after injection of sEphB4 showed a lower expression of VEGF, clearly indicating the connection of these signaling pathways.

Su et al. conducted studies in a murine model of laser-induced CNV. They showed that in animals, after intravitreal administration of EphB4 monoclonal antibody, CNV was smaller and thinner compared to the control group. Moreover, CNV progression was suppressed [78]. The above results show that there are substances other than anti-VEGF that can influence CNV formation. Since, as mentioned earlier, Eph and ephrins mainly modify the VEGF signaling pathways, there is a chance that combination therapy could help patients who do not or only respond poorly to treatment.

A summary of the identified associations of Eph and ephrins with CNV is presented in Table 4.

## 9. Therapeutic Possibilities

**Figure 6:**
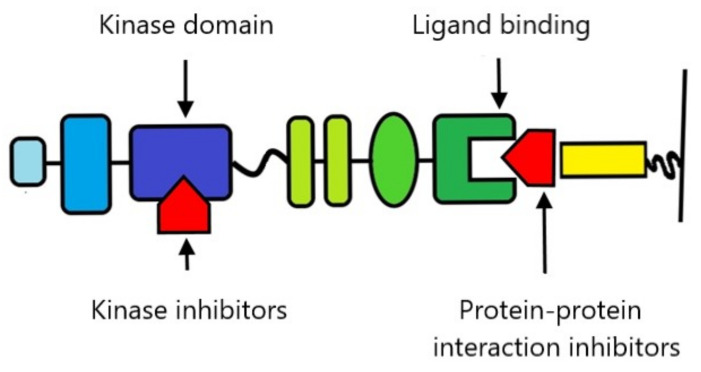
Schematic overview of the most important gripping points for Eph and ephrin therapy. Kinase inhibitors include nonselective compounds and second-generation selective inhibitors. Protein–protein inhibitors include antibodies, Eph and ephrin ectodomains, peptides, and small molecules.

In recent years, there has been growing interest in the possibility of using Eph and ephrins as therapeutic targets [79,80]. Their key functions for physiological and pathological tissue homeostasis make them an extremely tempting subject of research. Unfortunately, the complexity of Eph and ephrin reactions is also a source of additional challenges and difficulties, as the potential toxicity of drugs to the whole organism requires a very precise adjustment of the mechanism of action of drugs to the current needs. Research to date has focused on the two most obvious targets for therapy-blocking kinase activity and blocking the ligand-binding domain (Figure 6).

### 9.1. Kinase Inhibitors

Several tyrosine kinase inhibitors are currently in the clinical trials phase, which, among other things, are active against Eph. EXEL-7647, an inhibitor of EGFR, HER2, VEGFR2 and EphB4, demonstrated activity against non-small cell lung cancer in phase I and II clinical trials. Dasatinib, a drug approved for the treatment of chronic myeloid leukemia, is currently being tested in clinical trials in patients with cutaneous melanoma, glioblastoma multiforme, endometrial, prostate and lung cancer [81]. It has been shown to block, inter alia, the activity of EphA2, EphB1, EphB2 and EphB4. Other drugs that have shown activity against Eph under laboratory conditions and are entering clinical trials are nilotinib (inhibition of EphB2 and EphB4), bosutinib (inhibition (EphB4) and bafetinib (inhibition of EphA2, EphA5 and EphA8) [82]. A separate group are new generation inhibitors designed to selectively bind to Eph, for example, NVP-BHG712, which blocks the autophosphorylation of EphB4. In studies in mice, it has been shown to inhibit angiogenesis when administered orally [83].

### 9.2. Small Molecules

The formation of particles capable of influencing protein–protein reactions is a challenge because the protein–protein interface is much larger than that of a protein-small molecule. However, numerous attempts are made due to the enormous amount of potential substances that can be used. The most promising preparations acting on Eph include lithocholic acid derivatives (including UniPR126, which prevents EphA2 activation), salicylic acid derivatives (their potential to deliver other substances to Eph is mainly studied), green tea polyphenols, and their metabolites (their use is mainly limited by poor stability), doxazosin (a selective α1-adrenergic receptor inhibitor, which in mice studies has shown the ability to reduce prostate cancer metastasis and prolong survival) and peptide analogs mimicking the GH loop of ephrin [15].

### 9.3. Peptides, Peptide Analogs and Proteins

The G-H loop, i.e., 15 consecutive amino acids in the ephrin sequence, is responsible for binding to Eph. It was possible to isolate a sequence of 12 amino acids, which, under laboratory conditions, shows a high Eph blocking capacity. And although each of the ephrins binds to a greater or lesser degree to most Eph of the same class, some of the peptides show remarkable selectivity, binding only to one receptor [84]. Numerous studies in mice have shown the effectiveness of soluble forms of recombinant proteins in cancer therapy, among others melanoma, breast, lung, prostate, and colon cancer [82].

### 9.4. Antibodies

There are numerous studies on recombinant anti-Eph antibodies. B11 antibody administered intravenously inhibits the growth of blood vessels. The MAb131 and MAb47 antibodies, by binding to the fibronectin type III motif in EphB4, reduce the size of solid tumors in mice. IgG25 and IgG28 by binding to EphA2 also reduce tumor size [82].

## 10. Safety of the Use of Drugs Acting on Eph in the Eyes

As we have shown above, numerous studies have confirmed the role of Eph receptors and ephrins in retinal diseases, particularly those related to the formation of abnormal blood vessels, fibrous-vascular proliferation, and scarring. Expression of Eph and ephrins within retina under pathological conditions makes them a potentially attractive target for therapy. There are several clinical trials in which patients are given systemic EphB inhibitors (clinical trial numbers: NCT02717156; NCT02767921; NCT03049618; NCT03146971; NCT02495896; NCT01642342; NCT03049618). The results have yet to be published, but none of the studies have been discontinued due to unacceptable toxicity.

It should be remembered that in ophthalmology, drugs are mainly used topically. In the case of retinal diseases, the preferred route of administration is injection: intravitreal, possibly periocular, or under Tenon’s capsule. Drops administered into the conjunctival sac are much less common because the penetration into the eye of the drug administered in this way is extremely poor. Therefore, the potential toxicity to the structures of the eyeball will be of decisive importance. The retina in particular is sensitive to potential toxic damage [85]. Under the influence of various substances administered to the eye, necrotic changes in the retina, damage to photoreceptors, clouding of the lens or even its dissolution, and damage to the cornea, its clouding or accumulation of deposits can occur. Toxicity is usually related to the concentration of a substance.

In the previously described studies, EphB inhibitors were administered by intravitreal injections to mice and rats [61,62,63,86]. The researchers did not describe any significant side effects. Of course, they focused primarily on the test substances’ anti-angiogenic activity, but these studies analyzed retinal immunohistochemical preparations, so any necrotic changes would certainly be noted, as they would significantly affect the interpretation of the results. Unfortunately, the results of studies on small animals do not fully correspond to the conditions that prevail in human tissues. One reason is that in small animals the lens takes up much more of the eyeball volume than in humans. These values are over 32% in mice, 39% in rats and 3.5% in humans [87,88]. Such significant discrepancies result in changes in the distribution of the drug administered into the vitreous chamber, making it more difficult to transfer potential toxicity results to the human eye. For this reason, rabbits with a lens volume of about 16% are considered a better model for the human eye. Brar et al. in their study used rabbits [89]. They showed that intravitreal sEphB4 showed no toxicity to the eye tissues. They did not observe any opacities or inflammation, showed no abnormalities in electrophysiological tests, and did not find necrotic changes in the retina. The substances used were not detected in the plasma of the tested animals. It is worth emphasizing that the study used doses of the drug much higher than those that showed anti-angiogenic effectiveness in previous studies.

The drug’s specific half-life in the vitreous and retina is also of interest, particularly with respect to the anti-VEGF drugs used so far [89]. The vitreous half-life was 4.1 days for sEphB4, 2.84 to 3.2 days for ranibizumab (Lucentis) and 4.32 days for bevacizumab (Avastin). The calculated retina mean residence time was 10.45 days for sEphB4, 4.03 days for ranibizumab and 5.92 days for bevacizumab [90,91,92]. It was also shown that the mean residence time of the drug in the choroid was 7.95 days, which indicates good penetration of the drug through the retina. The good distribution profile of the drug within the eyeball is also evidenced by the substance’s comparable concentration in the vitreous body and the retina. Of course, Eph inhibitors’ precise safety profile still requires further intensive research, but the preliminary results are very promising.

Despite many difficulties, more and more centers are conducting research on the possibility of using drugs that affect the Eph and ephrin signaling pathways [24,93,94]. Over time, there should be more evidence of their safety, also for the retina and other eye tissues.

## 11. Summary

Blood vessel dysfunction, neovascularization, and the uncontrolled proliferation of fibroblasts underlie many retinal diseases. Among the most important are diabetic retinopathy, retinopathy of prematurity, age-related macular degeneration (AMD), proliferative vitreoretinopathy (PVR), and complications following the closure of large blood vessels in the retina. These diseases pose many therapeutic problems, and despite treatment they often progress, leading to a significant deterioration in visual acuity. Each of them conducts research on understanding pathophysiology to find new, more effective treatment methods. Therefore, the Eph receptors and ephrin associated with VEGF, participating in angiogenesis, migration, proliferation, and affecting the vessels walls’ stabilization, constitute an exciting subject of research for ophthalmologists. EphrinA1, ephrinA4, ephrinA5, EphA2, EphA7, ephrinB2, EphB3, and EphB4 seem to be the most important in the context of retinal diseases. However, it seems that further research may also show a significant role for other Eph and ephrins.

Particularly promising in the context of understanding the pathophysiology of retinal diseases are studies showing that by influencing Eph function, pathological angiogenesis can be inhibited without disturbing the development of normal blood vessels. This discovery, the most important in the context of ROP, may also facilitate the understanding of the detailed mechanisms of blood supply disorders within the retina.

On the other hand, in the context of treatment, studies showing the correlation between Eph/ephrin and VEGF signaling seem to be the most important. Anti-VEGF therapies are already the gold standard in ophthalmology. However, as the response to treatment is insufficient in some patients, the discovery of new therapeutic options is extremely promising. The safety profile of the substances affecting Eph, their bioavailability when applied to the eye, and their excellent penetration into the retina appear even more exciting. Much research is still needed before potential drugs could be widely used in ophthalmology, but it is worth making efforts to be able to treat patients even better in the future.

## Figures and Tables

**Table 1:** A summary of the identified associations of Eph and ephrins with diabetic retinopathy.

Name	Established Association with Diabetic Retinopathy	Possible Mechanism	Possible Use in Clinical Practice	Reference
ephrinA1	Increased plasma levels	Possible association with the pathogenesis of diabetic retinopathy	Diagnostic possibilities in the early stages of diabetic retinopathy	[50]
ephrinA1	Reduction of neovascularization following intravitreal administration	Inhibition of VEGFR2 receptor phosphorylation and suppression of multiple downstream signaling cascades (PKC-ERK1/2 pathway and Akt)	Potential therapeutic target	[51]
ephrinA1	Reduction of retinal vascular leakage following intravitreal administration	Inhibition of VEGF and the VEGF-dependent PKC signaling pathway in the retina	Potential therapeutic target	[51]
ephrinB2	Increased levels in retinal tissue with diabetic retinopathy	Involvement in ADMA-dependent angiogenesis	Potential therapeutic target	[52]
ephrinB2 and EphB4	Increased expression in neovascular membranes obtained from patients with diabetes compared to healthy tissues	Possible association with the pathogenesis of diabetic retinopathy	Potential therapeutic targets and biomarkers for diagnosis	[53]
ephrinB2, EphB2, and EphB3	Expression in fibroproliferative membranes obtained from patients with diabetic retinopathy	Involvement in ocular angiogenesis, stabilization and maturation of blood vessels	Potential therapeutic targets	[54]

**Table 2:** A summary of the identified associations of Eph and ephrins with retinopathy of prematurity.

Name	Established Association with Retinopathy of Prematurity	Possible Mechanism	Possible Use in Clinical Practice	Reference
ephrinA4	Overexpression in the retina with Neovascularization Reduction in the number of neovascular tufts following intravitreal injection of an inhibitor (blocking ephrinA4)	Involvement in neovascularization	Potential therapeutic target	[59]
ephrinA5	Overexpression in the retina with neovascularization	Involvement in neovascularization	Potential therapeutic target	[60]
EphA2	Blocking EphA2 reduces pathological neovascularization without affecting the formation of normal retinal vessels.	EphA2 may be necessary for maximal induction of neovascularization by VEGF	Potential therapeutic target	[61]
ephrinB2 and EphB4	Reduction in the number of preretinal tufts following intravitreal injection of soluble forms of ephrinB2 and EphB4	Regulating the processes of retinal neovascularization	Potential therapeutic target	[62]
ephrinB2 and EphB4	Stimulation of EphB4 and ephrinB2 signaling enhanced hypoxia-induced angiogenesis	Regulating the processes of retinal neovascularization	Potential therapeutic targets	[64]

**Table 3:** A summary of the identified associations of Eph and ephrins with proliferative vitreoretinopathy.

Name	Established Association with Proliferative Vitreoretinopathy	Possible Mechanism	Possible Use in Clinical Practice	Reference
ephrinB2 and EphB4	Expression in PVR membranes	Effect on migration and transdifferentiation of RPE cells	Potential therapeutic target	[63]
EphB4	Reduction of PDGF-induced migration and proliferation of RPE cells by the soluble form of EphB4	Reduction in FAK and p42/44 MAPK dependent phosphorylation	Potential therapeutic target	[69]

**Table 4:** A summary of the identified associations of Eph and ephrins with choroidal neovascularization.

Name	Established Association with Choroidal Neovascularization	Possible Mechanism	Possible Use in Clinical Practice	Reference
EphA7	Expression in neovascular membranes	Possible association with the pathogenesis of CNV	Potential therapeutic target	[76]
ephrinB2 and EphB4	Expression in choroidal endothelial cells Reduction in the leakage score of CNV membranes following intravitreal administration of a soluble form of EphB4	Role in maintaining the stability of the vessels Inhibition of choroidal endothelial cells migration	Potential therapeutic target	[62]
EphB4	Reduction in the leakage score of CNV membranes following intravitreal administration of soluble form of EphB4 Reduction of VEGF following intravitreal administration of a soluble form of EphB4	Inhibition of choroidal endothelial cells migration Connection of VEGF and Eph signaling pathways	Potential therapeutic target	[77]
EphB4	Slowing the progression of CNV following intravitreal administration of EphB4 monoclonal antibody	Possible association with the pathogenesis of choroidal neovascularization	Potential therapeutic target	[78]
